# Evaluation of the Effect of a Grape Seed Tannin Extract on Wine Ester Release and Perception Using In Vitro and In Vivo Instrumental and Sensory Approaches

**DOI:** 10.3390/foods10010093

**Published:** 2021-01-05

**Authors:** Carolina Muñoz-González, Celia Criado, María Pérez-Jiménez, María Ángeles Pozo-Bayón

**Affiliations:** Instituto de Investigación en Ciencias de la Alimentación (CIAL), CSIC-UAM, C/Nicolás Cabrera, 9, 28049 Madrid, Spain; celia.criado@csic.es (C.C.); maria.perez@csic.es (M.P.-J.); m.delpozo@csic.es (M.Á.P.-B.)

**Keywords:** wine, tannins, volatile compounds, polyphenol-aroma interactions, saliva, in vitro release, in vivo release, retronasal aroma, time-intensity, HS-GC/MS

## Abstract

This study aimed to systematically evaluate the effect of a commercial grape seed tannin extract (GSE) fully characterized (53% monomers, 47% procyanidins) on wine ester release and perception using a global approach. The behavior of two esters (ethyl hexanoate, ethyl decanoate) was studied in a control wine or in the same wine supplemented with the GSE in preconsumption (in vitro headspace-stir bar sorptive extraction-gas chromatography mass spectrometry (HS-SBSE-GC/MS) and orthonasal perception) and consumption (intraoral-HS-SBSE-GC/MS and dynamic retronasal perception) conditions. For the compound ethyl hexanoate, no significant differences (*p* > 0.05) among wines were observed in the in vitro analyses while they were observed in the three in vivo experiments (*p* < 0.05). Thus, the wine supplemented with the GSE showed lower (35%) in vivo release and ortho (36%) and retronasal (16%) perception scores than the control wine. Overall, this suggests that components of the GSE could interact with this compound, directly and/or through complexes with oral components, affecting its release and conditioning its perception. However, perceptual interactions and effects of polyphenols on oral esterases cannot be discarded. On the contrary, the compound ethyl decanoate was not significantly affected by the addition of GSE. In conclusion, the addition of tannin extracts to wines can modulate aroma perception in a compound-dependent manner.

## 1. Introduction

Grape polyphenols can be naturally present in wines, or they can be added as oenological extracts. Grape tannin extracts usually contain condensed tannins (or proanthocyanidins) and other components, depending on their purity [1]. Their addition to wines was related to providing color stabilization, contributing to wine structure and mouthfeel [1,2] or to wine flavor [3], among others. Apart from their effects on astringency and bitterness [3], recent studies have suggested that grape tannin extracts could modulate wine aroma [4,5], which is one of the main drivers for wine preferences [6].

The mechanisms behind the modulator effect of grape tannin extracts on wine aroma are unknown, but different hypotheses were proposed in the literature. On the one hand, the capacity of components present on the tannin extracts to interact with some volatile compounds was proven using in vitro conditions in model wines [7,8,9]. In 1999, Dufour and Bayonove [7] observed that the addition of a condensed tannin fraction (0–14 g/L) to a hydroalcoholic solution provoked a small reduction of benzaldehyde volatility and an increase of limonene volatility while had no effect on two esters (isoamyl acetate, ethyl hexanoate). Accordingly, Mitropoulou and coworkers [8] observed that the addition of a condensed tannin extract (0–12 g/L) to a model wine had almost no influence on the volatility of some esters (isoamyl acetate and ethyl hexanoate) while others (ethyl octanoate, ethyl decanoate, ethyl dodecanoate) suffer a salting-out or retention effect depending on the tannin concentration assayed. In 2013, Villamor and coworkers [9] found that increasing tannin addition (500 mg/L–1500 mg/L) to a model wine increased the release of high molecular weight compounds to the headspace (1-octen-3-one, 2-methoxyphenyl, eugenol) but decreased the release of alcohols (3-methyl-1-butanol, 1-hexanol). From these studies, it could be presumed that these interactions could affect the availability of the aroma compounds to reach the olfactory receptors through the orthonasal pathway, affecting most likely wine odor [9]. However, olfaction is a complex phenomenon that can also be affected by several biases, such as the way sniffing is performed ([10]), and therefore, studies are needed to verify this point.

During consumption, wine is introduced in the oral cavity and components of the tannin extracts can interact with salivary proteins in the mouth (free or bound to the mucosal pellicle) [11,12,13,14] or directly to the epithelial mucosa cells [15]. As side effects, these interactions may influence the oral retention of volatiles and their transfer to the olfactory receptors through the retronasal pathway. Thus, it was suggested that some polyphenols already adsorbed onto the mucosal pellicle might bind certain aroma molecules with aromatic rings, such as guaiacol [16], increasing their retention in the mouth. Apart from physicochemical interactions, polyphenols present in the tannin extracts could affect the activity of salivary enzymes [17]. This would be of special relevance in the case of enzymes involved in the metabolization of aroma compounds [18]. In this regard, inhibition of esterases by grape seed tannin extracts was recently suggested [4,17]. Thus, the influence of tannin extracts on salivary enzymes deserves more research. Finally, aroma perception during wine consumption is a complex phenomenon produced as a result of the integration in the brain of multiple sensory signals [19,20]. Therefore, to understand the meaning of the polyphenol-volatile interactions in a real consumption context, wine retronasal aroma perception must be assessed.

Additionally, it is important to notice that the technical effects provided by the tannin extracts will be dependent upon their composition. In this regard, there is a wide range of oenological extracts in the market, although, in general, there is a lack of information about their composition [1]. This makes it a challenge to attribute the effects of these extracts to specific components, making it difficult the comparison across studies. The aim of this work was to systematically evaluate the effect of a grape seed tannin extract (GSE) previously characterized [21] on the release and perception of two target volatile compounds using in vitro and in vivo instrumental and sensory approaches. To do this, a wine with a low-aroma profile was enriched with two esters (20 mg/L), which are typical ubiquitous volatile compounds from alcoholic fermentation and relevant compounds contributing to the wine aroma profile. The aromatized rosé wine (control wine) was supplemented with the GSE (at 500 mg/L). Ester release was studied in vitro, through headspace-stir bar sorptive extraction-gas chromatography mass spectrometry (HS-SBSE-GC/MS), and in vivo, through intra-oral-HS-SBSE-GC/MS (*n* = 10). Additionally, orthonasal and the dynamics of retronasal aroma perception during wine tasting were evaluated through sensory evaluation (*n* = 10). The same panelists participated in all the experiments. To our knowledge, this is the first time that such a global approach was applied to understand the effect of oenological tannins on wine aroma.

## 2. Materials and Methods

### 2.1. Wine Samples

A commercial rosé wine (D.O. Navarra) (pH of 3.03, 13% *v*/*v* ethanol, total polyphenol content of 355.88 ± 1.74 mg Eq Gallic acid/L and procyanidin content of 66.37 ± 0.01 mg/L) was selected for this study by its low polyphenol content. A commercial tannin extract from grape seeds (GSE) (Vitaflavan^®^) (D.R.T. Les Dèrives Resiniques and Terpéniques, Vielle-Saint-Girons, France) mainly composed of 53% monomers and 47% procyanidins was added to this control wine (CW) at a concentration of 500 mg/L, to obtain another wine with a higher polyphenol content (PW). The individual phenolic composition of this extract was previously published [21]. The GSE was deodorized under an N_2_ current for 30 min in order to remove endogenous aromas.

Two food-grade linear esters (Sigma-Aldrich, Steinheim, Germany) with different physicochemical properties (Table 1) were added to the wine glasses to obtain a final concentration of 20 mg/L. The technique HS-HSSE-GC/MS [22] was used to evaluate the presence of endogenous aroma compounds in the original wine that was insignificant compared to the concentration of the aroma added (ethyl hexanoate: 0.37 ± 0.02 mg/L; ethyl decanoate: not detected).

### 2.2. Panel

For this study, ten young individuals (8 females, 2 males) were recruited. A triangular test with aromatized hydroalcoholic solutions was performed to evaluate their absence of anosmia. All the individuals received information on the nature of the study and provided written consent form before their participation. They were instructed not to eat or drink one hour before the instrumental or sensory experiments. The CSIC Bioethics Committee for Research approved the experimental protocol.

### 2.3. Instrumental Evaluation

#### 2.3.1. In Vitro Ester Release

A previous method [22] was followed with slight modifications. Briefly, wine samples (1-mL) were placed in headspace vials (20-mL) containing a glass insert with a preconditioned PDMS (polydimethylsiloxane) stir bar (Twister) (Gerstel, Mülheim, Germany). Extraction was performed for 15 min, and then, the twisters were removed for thermal desorption. The experiments were done in triplicate.

#### 2.3.2. In Vivo Ester Release

An instrumental procedure developed by Pérez-Jimenez and Pozo-Bayón [22] was used. Briefly, each individual introduced the wine samples (15 mL) into their mouths, kept them for 30 s and then expectorated them. Two aroma extractions were performed. The first one, five seconds after expectoration, and the second one, sixty seconds later. The extractions were performed for 30 s with a PDMS twister (Gerstel, Mülheim, Germany) introduced in the oral cavity using glass made twister holders. Once the extraction was finished, the twisters were removed for thermal desorption. Each wine sample was analyzed in triplicate by each individual. Controls of the oral cavity before wine rinsing were also performed to ensure the absence of the esters under study.

#### 2.3.3. Thermal Desorption and Gas Chromatography Mass Spectrometry (GC–MS) Analyses

The twisters were desorbed using a TDU, and a CIS-4 injector (Gerstel, Mülheim, Germany) coupled to a 6890 N GC—5973 N mass spectrometers (Agilent). Compounds were separated in a DB-WAX column (30 m × 0.25 mm i.d. × 0.50 μm film thickness) (J&W Scientific, Folsom, CA, USA) using the following oven temperatures: 40 °C, held 1 min, 10 °C/min to 240 °C and 240 °C held 1 min (run time: 18 min). The acquisitions were performed in the scan (from *m*/*z* 35 to 350) and SIM modes. For specific details on the GC–MS conditions, see Pérez-Jimenez and Pozo-Bayón [22].

Aroma identification was done by using retention times and mass spectra provided by the NIST 2.0 database. The aroma release between wine samples was done on the basis of absolute peak areas (APAs).

### 2.4. Sensory Evaluation

#### 2.4.1. Training Sessions

First, individuals were trained to recognize orthonasally and retronasally the two aroma descriptors of the study (fruity and grape) in a wine matrix containing the respective chemical references (ethyl hexanoate and ethyl decanoate). Second, they received specific training on the use of the intensity scale (15 cm unstructured scale delimited at the ends) and on the discrimination between different aroma intensities (corresponding to 1, 3, 9 and 27 mg/L of each aroma compound). Third, individuals were trained in the protocol for wine consumption to evaluate retronasal aroma perception and in the use of tablet devices using the Compusense software for time-intensity (TI) measurements. Six training sessions were performed by all the individuals, while three of them received 2 additional sessions to be fully trained. The serving temperature of the samples was 18 °C. Samples were served in wine glasses covered with plastic Petri dishes to prevent volatile loss. Water (Nestle Aquarel, Barcelona, Spain) and unflavored unsalted crackers (ARO, Madrid, Spain) were provided to the individuals among samples.

#### 2.4.2. Orthonasal Evaluation

Wine samples were equilibrated for 15 min. Then, individuals were instructed to remove the plastic Petri dishes over the wine glasses, to smell them and to evaluate the intensity of the two descriptors (fruity and grape) using an unstructured scale. Samples were evaluated in triplicate and in randomized order.

#### 2.4.3. Retronasal Evaluation by Time-Intensity (TI)

Individuals were trained to follow the instructions: introduce the wine samples (15 mL) into their mouths and expectorated them after 5 s. After this time, individuals started the TI evaluation of the two aroma descriptors (fruity and grape) using the Compusense software (version 19.0.7236.30304, Guelph, Canada) that lasted 60 s. To do so, they had to move the cursor along the unstructured scale (15 cm) to note the intensity of aroma descriptors perceived. Data were recorded at a frequency of 1 s. Samples were evaluated in triplicate and in randomized order.

Mean curves of dynamic intensity scores for each wine were determined by averaging the data of all the individuals at each time point.

### 2.5. Statistical Analyses

One-way analysis of variance (ANOVA) and Tukey’s test for mean comparison were used to check the influence of the GSE on the in vitro and in vivo ester release and on the orthonasal and retronasal ester perception. The significance level was *p* < 0.05, α < 0.05 throughout the study. The XLSTAT program (v.19.01) (Addinsoft, Paris, France) was used for data processing.

## 3. Results

### 3.1. Effects of a Grape Seed Tannin Extract (GSE) on the *In Vitro* Ester Release from Wine

**Figure 1:**
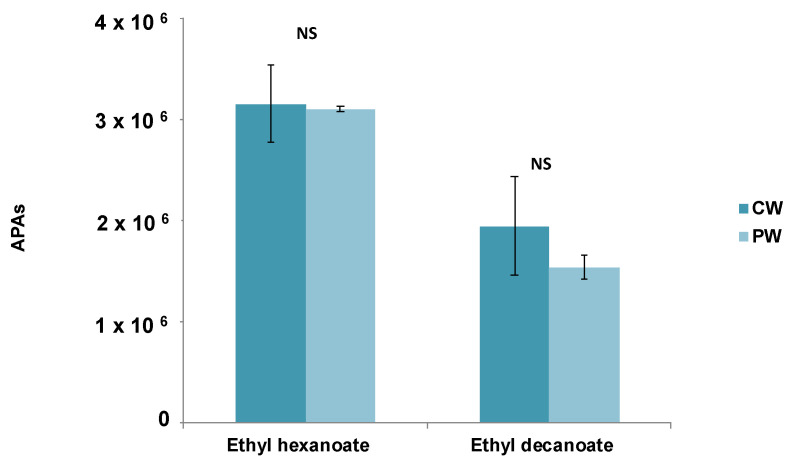
Comparison of the absolute peak areas (APAs) of esters released to the headspace in the control wine (CW) and in the wine with grape seed tannin extract (GSE) added (PW) using in vitro conditions (NS: no significant differences (*p* > 0.05) between wine samples).

To determine the effect of the GSE in preconsumption (in vitro) conditions, the APAs of esters recovered in the headspace above the CW and PW were submitted to a one-way ANOVA. The results are shown in Figure 1. As can be seen, no significant differences were observed between wines for any of the two esters assayed (*p* > 0.05). Although the release of both esters was slightly higher in the CW compared to the PW, these differences were not statistically significant (*p* > 0.05).

### 3.2. Effects of a Grape Seed Tannin Extract (GSE) on the *In Vivo* Oral Ester Release from Wine

**Figure 2:**
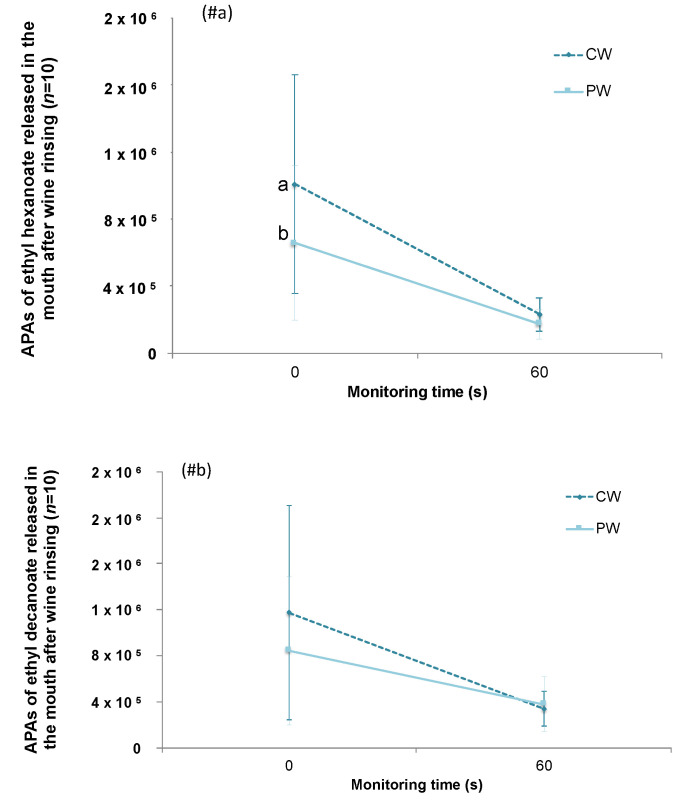
Comparison of the absolute peak areas (APAs) of ethyl hexanoate (**#a**) and ethyl decanoate (**#b**) released in the mouth after oral rinsing with the control wine (CW) and with the wine with the GSE added (PW) using in vivo conditions. Different letters across the different time points indicate significant differences between wines (*p* < 0.05, α < 0.05).

To check the influence of the GSE in consumption conditions, the esters released into the oral cavity of ten individuals after they rinsed their mouths with the two wines (CW and PW) was followed at two sampling points (0 and 60 s). The APAs of esters recovered intra-orally were submitted to a one-way ANOVA. The results can be observed in Figure 2.

As expected, a decrease in ester release was observed over time in the oral cavity, although the rate of the decrease seemed faster for the CW than for the PW. Moreover, large interindividual differences on the intra-oral ester release were observed for both compounds, mostly on the first monitoring time. The presence of the GSE seemed to decrease the intra-oral release of both esters. Although a similar trend was observed for both esters (Figure 2), results of the ANOVA test only showed a significant effect (*p* < 0.05) of the GSE for the compound ethyl hexanoate on the first monitoring time (0 s) (being a 35% less released in PW compared to CW). In the second monitoring time (60 s), no significant differences between wines were observed for any of the esters assayed.

### 3.3. Effects of a Grape Seed Tannin Extract (GSE) on the Orthonasal Perception of Wine Esters

**Figure 3:**
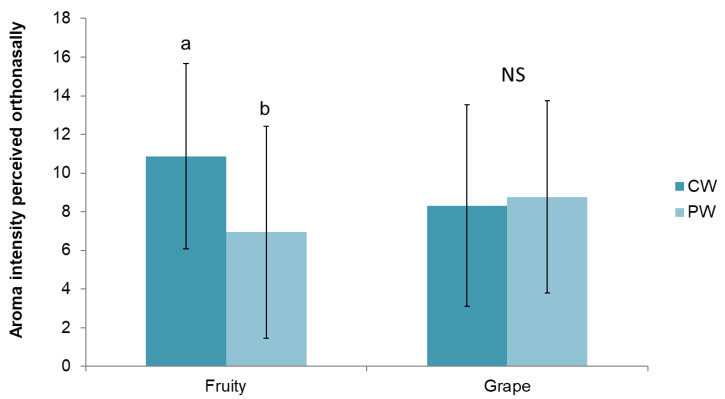
Intensity scores of the aroma descriptors determined orthonasally by the trained panel (*n* = 10) in the control wine (CW) and in the wine supplemented with the GSE (PW). Different letters across the different time points indicate significant differences between wines (*p* < 0.05, α < 0.05); NS: no significant differences between wines (*p* > 0.05).

A one-way ANOVA was performed to evaluate the effect of the GSE addition on the orthonasal perception of the two aroma descriptors (fruity and grape) by 10 individuals (Figure 3). The evaluation of the two aroma descriptors was done independently of one another.

As it can be seen, significant differences were observed in the perception of fruity note (from ethyl hexanoate) between wines, with PW showing a significantly lower score (36%) for fruity intensity than CW. In contrast, no significant differences were observed for the perception of grape note (ethyl decanoate) between wines.

### 3.4. Effects of a Grape Seed Tannin Extract (GSE) on the Retronasal Perception of Wine Esters

**Figure 4:**
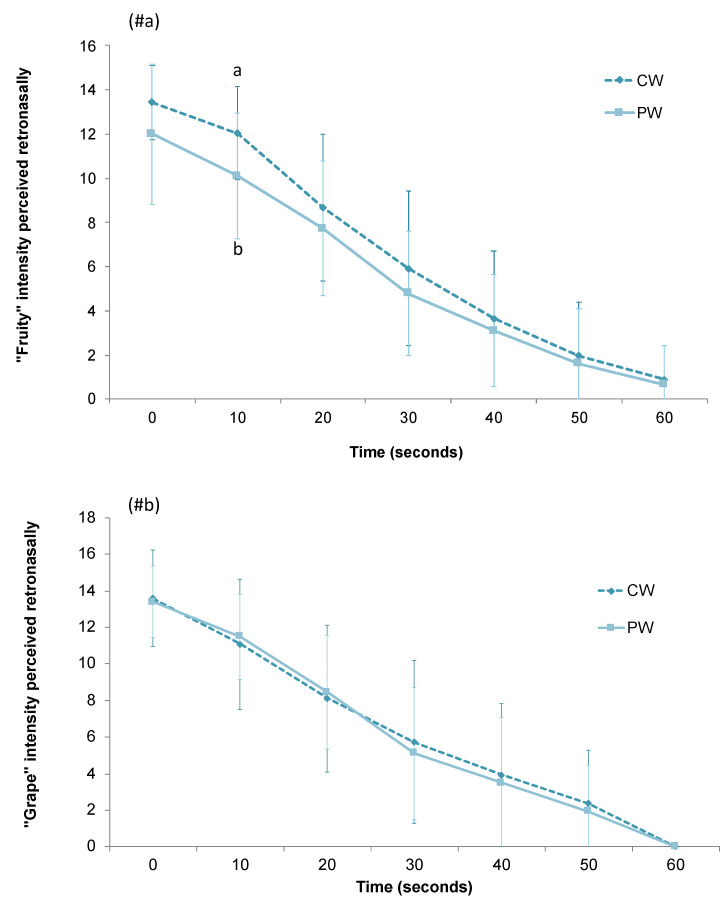
Intensity scores of the fruity (**#a**) and grape (**#b**) descriptors determined retronasally by the trained panel (*n* = 10) in the control wine (CW) and in the wine supplemented with the GSE (PW). Different letters across the different time points indicate significant differences between wines (*p* < 0.05, α < 0.05).

A one-way ANOVA was carried out to check the effect of the GSE addition on the perception of the aroma descriptors elicited by the two esters through a dynamic sensory TI evaluation. The evaluation of the two aroma descriptors was done independently of one another. As it can be seen (Figure 4), a decrease in the intensity notation of both attributes was observed over time. The GSE seemed to suppress the perception of the fruity attribute (ethyl hexanoate), while no effect was observed for the grape note (ethyl decanoate). However, the differences observed for the fruity note were only significant in the monitoring time of 10 s (Figure 4), with a difference in the intensity score between PW and CW of 16%.

## 4. Discussion

The effects of tannin extracts on wine aroma are hardly understood, among other reasons, because the comparison across studies is difficult. Usually, studies on this topic have employed commercial extracts of unknown/not specified composition, which generates complications to compare the results and to extract conclusions. Apart from that, the fact that the studies on this topic are made with different experimental protocols and/or techniques or with wines with a different chemical composition (volatile and matrix) adds a degree of uncertainty to this topic. For these reasons, a global and systematic approach was followed in this study. A grape seed tannin extract (GSE) (53% monomers, 47% procyanidins), fully characterized previously [21], was used to evaluate its influence on wine aroma release and perception. A rosé wine with (PW) or without (CW) the GSE was systematically assayed using a wide array of instrumental and sensory techniques. The wine was aromatized with two esters, ethyl hexanoate (fruity note) and ethyl decanoate (grape note), that were selected for being compounds from the same chemical family (esters) presenting different physicochemical properties (Table 1) and because they are associated with pleasant aroma notes easily recognizable (ortho and retronasally) by consumers. The same individuals (*n* = 10) participated in all in vivo (instrumental and sensory) experiments, and they were previously trained to follow specific tasting protocols. Before the experimentation, the absence of anosmia was checked for all the individuals in preliminary triangular tests.

The effects of the GSE on wine esters were firstly evaluated in pre-consumption conditions, that is, before the wine is introduced in the mouth. One of the first sensory signals perceived by wine consumers is related to its odor, produced when volatiles are released from the wine matrix and reach the olfactory receptors by the orthonasal pathway. This phenomenon was evaluated through two approaches: one instrumental by measuring the esters released to the headspace above the wines (CW, PW) in in vitro conditions, and one sensory approach by measuring the orthonasal perception of the individuals (*n* = 10) when smelling the wines. Results from the in vitro static headspace analyses did not show a significant effect (*p* > 0.05) of the GSE on the release of esters to the headspace (Figure 1). These results are in agreement with those from Dufour and Bayonove and Mitropoulou et al. [7,8], who found that the addition of a tannin extract to model wines did not affect the release of ethyl hexanoate at any of the assayed tannin extract concentrations. On the contrary, Mitroupoulu and collaborators [8] did observe a salting-out effect on ethyl decanoate upon tannin addition, which was not observed in the present study. Divergences among studies could be due to compositional differences among the extracts assayed and/or the concentrations employed (0.5 g/L vs. 0–12 g/L), although no direct comparison can be made since in work from Mitropoulou et al. [8] the composition of the tannin extract was not specified. Regarding the results of the orthonasal experiment (Figure 3), significant differences were observed between wines (CW and PW) for the fruity note provided by the compound ethyl hexanoate, while no effects were observed for the grape descriptor associated with the compound ethyl decanoate. Thus, the fruity note was less (36%) intensely rated by the individuals in the PW compared to the CW. Although to the authors’ knowledge, the effects of procyanidins on the orthonasal perception of wine esters has not been elucidated to date, the effects of monomers and, in particular, of catechin and gallic acid (also constituents of the grape seed extract) on the suppression of perception of esters was confirmed in several works [23,24,25]. Thus, this suppressed perception could be due to the monomers present in the extract, although the role of procyanidins cannot be discarded.

These apparently contradictory results between the instrumental and sensory experiment performed in preconsumption conditions were also observed by Lorrain and colleagues [23] when studying the effects of phenolic compounds on the volatility and sensory perception of red wine esters in model solutions. In that article, the authors found a significant effect of the compound catechin on the orthonasal perception of specific esters, although the same effects were much less clear on the headspace experiments. They related this contradiction to a conflict on sensitivity between mass spectrometry and the human nose, being the latter more sensitive than the former. Moreover, they stated that the weakness of the polyphenol-volatile hydrophobic interactions (also suggested by Dufour and Bayonove [7]) could have produced that small changes between instrumental and sensory approaches (volumes, temperatures, extraction times) would lead to a disruption of the interactions between polyphenols and volatiles. Overall, this could also have occurred in the present work.

During consumption, wine is introduced into the oral cavity. From here, aroma compounds interact with oral components before traveling to the olfactory receptors located in the nose to be perceived. In an attempt to decipher the effects of the GSE on wine aroma in a consumption situation, two complementary instrumental and sensory approaches were used. First, the esters released in the oral cavity of the individuals after wine rinsing were monitored through a previously developed technique called intraoral-HS-SBSE-GC/MS [22]. Figure 2 shows that the intra-oral ester release decreased over time, which means that esters disappeared from the oral cavity after wine rinsing, as was expected. Interestingly, the decrease rate seemed faster in CW than in PW, which could indicate that the GSE addition could have an effect on aroma persistence. This could be related to an inhibitory effect of polyphenols (present in the GSE) on salivary enzymes as it was recently suggested [17], which will deserve more research. Another interesting finding was related to the fact that the intra-oral release was significantly different among wines during the first monitoring time, with PW releasing less ethyl hexanoate to the oral headspace than CW (35% difference). This could be the consequence of the polyphenol-volatile interaction observed in the orthonasal analyses and would indicate that a lower amount of ethyl hexanoate molecules could be available to reach the olfactory receptors immediately after wine rinsing in PW than in CW. In addition, it could not be discarded that interactions between components of the GSE and oral components (salivary proteins, buccal cells) [8,9,16] could have affected, as side effects, ethyl hexanoate volatility. In this regard, a negative relationship between flavan-3-ol content in wines and intra-oral aroma release of esters, and especially of ethyl hexanoate, was previously observed [16]. In that study, it was suggested that this phenomenon could be due to the formation of salivary proteins-polyphenol-carbohydrate complexes able to encapsulate aroma compounds in the oral cavity. On the contrary, for the compound ethyl decanoate, no differences between wines were found for any of the time points assayed. This result differed from that found by Muñoz-Gonzalez and coworkers [4], who observed an increase in the oral volatility of this compound after drinking wine with a similar extract added. Nevertheless, it should be noticed that the monitoring times and the concentration of the extract assayed were very different in both experiments, which confirms that the experimental conditions employed are determinant to compare the effects of GSE on wine aroma.

Second, the retronasal perception of the individuals after rinsing their mouths with the two wines was evaluated using the dynamic TI approach. In this case (Figure 4), the individuals rated with lower scores the fruity descriptor (ethyl hexanoate) in the PW compared to the CW, although significant differences were only observed at one evaluation time (10 s) (16% difference), maybe due to the high inter-individual differences observed. As in the case of the in vivo release experiments, no significant differences between wines were observed for the compound ethyl decanoate. These results are in agreement with those of Cliff et al. [26] that found suppression of fruity aroma in wine in the presence of a GSE (0–5 g/L). Similarly, Pérez-Jimenez and colleagues [5] observed a lower score of fruity notes (derived from esters) in wines with a GSE added (150 mg/L). The lower intensity of fruity perception observed for PW in comparison to CW could be related to the lower intra-oral release observed for this wine, derived from polyphenol-volatile or polyphenol-oral components-volatile interactions. However, it is important to highlight that during wine drinking, many senses are activated at a time which would inevitably modulate the perceived intensities of each other through cross-modal sensory interactions. Taking into account the taste-aroma interactions in the process of drinking wine [27], the bitterness and astringency brought by polyphenols contained in the GSE may have influenced the perception of aroma. Thus, it could not be discarded that this phenomenon could have had an effect on the suppression of the fruity descriptor when it was retronasally perceived.

## 5. Conclusions

This research work has demonstrated that the addition of a GSE to a rosé wine influenced the release and perception of esters in a compound- dependent manner. For the compound ethyl hexanoate, the addition of the GSE did not significantly affect its release from wine measured in in vitro (headspace) conditions. However, the addition of the GSE decreased the in vivo release of ethyl hexanoate after wine rinsing measured intra-orally. Moreover, the addition of the GSE affected the orthonasal and retronasal fruity perception associated with this compound. Thus, participants scored with a lower fruity intensity the wine with the GSE added compared to the control wine. This suppression observed by using instrumental and sensory approaches suggests that some components of the extract (procyanidins and/or monomers of catechin, epicatechin, …) can interact directly and/or through complexes with oral components with the compound ethyl hexanoate. Apart from the physicochemical nature of these interactions, the existence of other phenomena such as perceptual interactions or some effects of polyphenols on esterase activity cannot be discarded. For the compound ethyl decanoate, no effects were observed due to the GSE addition.

## Figures and Tables

**Table 1:** Characteristics of the esters added to the wines.

Compounds	CAS Number	Physicochemical Characteristics				Sensory Descriptor ^d^
		Chemical Formula	Mw ^a^	*log P* ^b^	BP ^c^	
Ethyl hexanoate	123-66-0	C_8_H_16_O_2_	144	2.83	167	Fruity
Ethyl decanoate	110-38-3	C_12_H_24_O_2_	200	4.79	248	Grape

^a^ Molecular weight (g/mol). ^b^ Hydrophobic constant estimated with EPI suite (US EPA 2000–2007). ^c^ Boiling point (°C) estimated with EPI Suite (US EPA 2000–2007). ^d^ Flavornet (http://www.flavornet.org) database.

## Data Availability

The data presented in this study are available on request from the corresponding author. The data are not publicly available due to ethical reasons.
